# WCx-Supported RuNi Single Atoms for Electrocatalytic Oxygen Evolution

**DOI:** 10.3390/molecules28207040

**Published:** 2023-10-12

**Authors:** Jirong Bai, Yaoyao Deng, Yuebin Lian, Quanfa Zhou, Chunyong Zhang, Yaqiong Su

**Affiliations:** 1 Research Center of Secondary Resources and Environment, School of Chemical Engineering and Materials, Changzhou Institute of Technology, Changzhou 213022, China; baijr@czu.cn (J.B.); dengyy@czu.cn (Y.D.); lianyb@czu.cn (Y.L.); labzqf@czu.cn (Q.Z.); 2 School of Chemistry and Environmental Engineering, Jiangsu University of Technology, Changzhou 213001, China; 3 School of Chemistry, Xi’an Key Laboratory of Sustainable Energy Materials Chemistry, State Key Laboratory of Electrical Insulation and Power Equipment, Xi’an Jiaotong University, Xi’an 710049, China

**Keywords:** tungsten carbide, single-atom site, RuNi, oxygen evolution reaction

## Abstract

Single-atom catalysts anchored to oxide or carbonaceous substances are typically tightly coordinated by oxygen or heteroatoms, which certainly impact their electronic structure and coordination environment, thereby affecting their catalytic activity. In this study, we prepared a stable oxygen evolution reaction (OER) catalyst on tungsten carbide using a simple pyrolysis method. The unique structure of tungsten carbide allows the atomic RuNi catalytic site to weakly bond to the surface W and C atoms. XRD patterns and HRTEM images of the WCx-RuNi showed the characteristics of phase-pure WC and W_2_C, and the absence of nanoparticles. Combined with XPS, the atomic dispersion of Ru/Ni in the catalyst was confirmed. The catalyst exhibits excellent catalytic ability, with a low overpotential of 330 mV at 50 mA/cm^2^ in 1 m KOH solutions, and demonstrates high long-term stability. This high OER activity is ascribed to the synergistic action of metal Ru/Ni atoms with double monomers. The addition of Ni increases the state density of WCx-RuNi near the Fermi level, promoting the adsorption of oxygen-containing intermediates and enhancing electron exchange. The larger proximity of the d band center to the Fermi level suggests a strong interaction between the d electrons and the valence or conduction band, facilitating charge transfer. Our research offers a promising avenue for reasonable utilization of inexpensive and durable WCx carrier-supported metal single-atom catalysts for electrochemical catalysis.

## 1. Introduction

Proton exchange membrane hydrolysis pools have gained significant attention as an attractive avenue for large-scale sustainable hydrogen energy production [1,2,3,4,5]. Compared with the hydrogen evolution reaction (HER) on the cathode side, the oxygen evolution reaction (OER) on the anode side, including the transfer of four electrons, is considered to be the bottleneck of this technology, and is the key reaction in improving the electrolysis of hydrogen production [6,7,8,9,10,11,12]. This process calls for two steps, viz. O-H breakage and O-O formation, which result in a slow kinetic reaction and the need for a large overpotential to reach the necessary current density (≥10 mA/cm^2^). The generation of cheap and effective OER electrocatalysts to further reduce electrode overpotential is a major challenge [13,14,15,16,17]. For decades, a wide range of OER catalysts with low overpotentials have been reported to have replaced expensive precious metal electrocatalysts. Among them, single-atom catalysts have great potential because of their highest atom use efficiency and excellent catalytic activity, thanks to the atomically dispersed active centers [18,19,20,21,22].

According to the volcanic distribution of the binding energy of oxygen intermediates, Ru-based OER catalysts are among the main materials with suitable activity and stability. Optimization of Ru through atomical dispersion not only significantly improves the atomic utilization efficiency, but also greatly reduces the reliance on precious metals, thus alleviating cost concerns. As reported, an atomically dispersed Ru-N_4_ site installed on a N-C support (Ru-N-C) is an effective and durable electrocatalyst for acidic OER with a low overpotential of 267 mV and excellent stability at a current density of 10 mA/cm^2^ [23]. In addition, dual-atom catalysts with outstanding catalytic performance have been developed, such as RuCo [24]. 

However, single-atom OER catalysts tend to be attached to various carbon supports, forming a strong coordination environment with impure atoms (e.g., oxygen, nitrogen, and sulfur) through the ligand effect. This coordination environment is highly correlated with the binding energy of the adsorbent and significantly influences the electronic distribution of metal atoms and the activity of the single-atom active center. Unfortunately, this strong coordination environment hinders the synergy between dual atoms and is not conducive to OERs. Additionally, carbon-based support materials are prone to slow oxidation at high potential (>1.8 V), leading to reduced durability of the catalyst under high overpotential. Therefore, developing support materials with a well-defined structure, high electrical conductivity, excellent durability for OERs, and stable catalytic metal single atoms without strong coordination to heteroatoms is of utmost importance.

The use of transition metal carbides (TMCs) as carriers for loading metals or metal oxides has gained significant attention, owing to their excellent conductivity and stability [25,26,27,28,29]. Owing to the metal-like properties of the WCx substrate, the weak coordination between the single atom and the W/C atoms on the surface of WCx generates a low-state catalytic active site similar to the metal. Through the unique metal–metal interaction, the low-state atomic site is stabilized on the surface of WCx, and the electron orbital energy distribution of the catalytic active site is improved. To address this challenge, we are inspired by these studies and propose the use of WCx as a support material to stabilize multivariate single atoms of Ru-based catalysts (e.g., RuNi, RuMn, and RuCo), so as to develop advanced OER catalysts with high conversion efficiency. Our main motivation stems from the special metal-like electronic properties of WCx, which promote electron interactions that resemble metal–metal bonds between the supported metal atoms and the carbide surface. Consequently, the WCx support effectively stabilizes Ru and Fe/Ni/Co/Mn on the surface of the WCx carrier by forming Ru/M-W or Ru/M-C bonds. Through heat treatment, we successfully obtained NiRu dual-single atoms supported by tungsten carbide nanocrystals, which exhibited remarkable OER catalytic activity with an overpotential of 330 mV at 50 mA/cm^2^. 

## 2. Results and Discussion

### 2.1. Synthesis and Characterisation

**Scheme 1:**
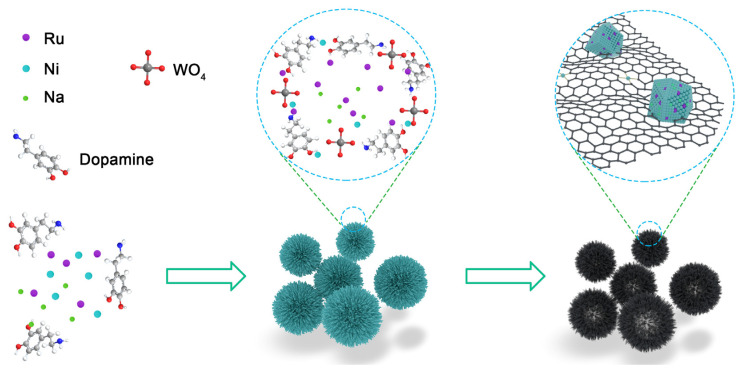
Schematic illustration of the synthetic procedures.

We utilized N- and O-containing organic molecules with strong complexation ability to assemble metal atoms and tungstate (${WO}_{4}^{2-}$) atoms, as bonding units with various metal salts through a precipitation reaction (Scheme 1). As a result, precursors with a uniform structure were formed. Subsequently, the OER catalyst based on the tungsten carbide nanocrystal support was obtained by heating the precursors at 900 °C for 2 h, forming aggregated spherical nanomaterials. Initially, we analyzed the morphology and surface structure characteristics of the WCx-RuNi catalyst and its precursors. Dopamine molecules coordinate with Ru and Ni ions, which then assemble with tungstate ions to form metal–organic compounds as powders. Figure S1a,b depicts the SEM images of the precursors (DA-WCx-RuNi), which exhibit microspherical shapes formed from aggregated nanosheets or flakes. Clearly, the nanomaterials possess various shapes, and the particles are relatively dispersed, but not in a regular or orderly arrangement. Moreover, the SEM image of the WCx-NiRu catalyst after heat treatment shows a microspherical morphology similar to that of the precursor, but with a wrinkled and uneven surface (Figure S1c,d). This surface structure provides additional active reaction sites, which promote the reaction and enhance catalytic efficiency. The crystal structures of the WCx-NiRu catalysts were analyzed using powder X-ray diffraction (XRD). Figure S2 shows the peaks corresponding to both WC and W_2_C phases, implying that two evident tungsten-carbide-based phases coexist. This observation was made by comparing the XRD patterns of the WCx-RuNi catalysts with those of phase-pure WC and W_2_C samples [25,30,31]. The typical peaks at 31.63, 35.80, and 48.52° are assigned to the (001), (100), and (101) planes of hexagonal tungsten carbide, respectively (WC, JCPDS No. 51-0939) [32]. Relative to the peaks of WC, some of the lower-intensity peaks at 34.50, 38.20, 39.57, and 52.38° correspond to W_2_C of (001), (100), and (101) planes of simple hexagonal phase, respectively (JCPDS No. 35-0776) [33,34,35]. In addition, no significant peak attributed to RuNi alloy was observed, which was probably due to its low concentration. 

**Figure 1:**
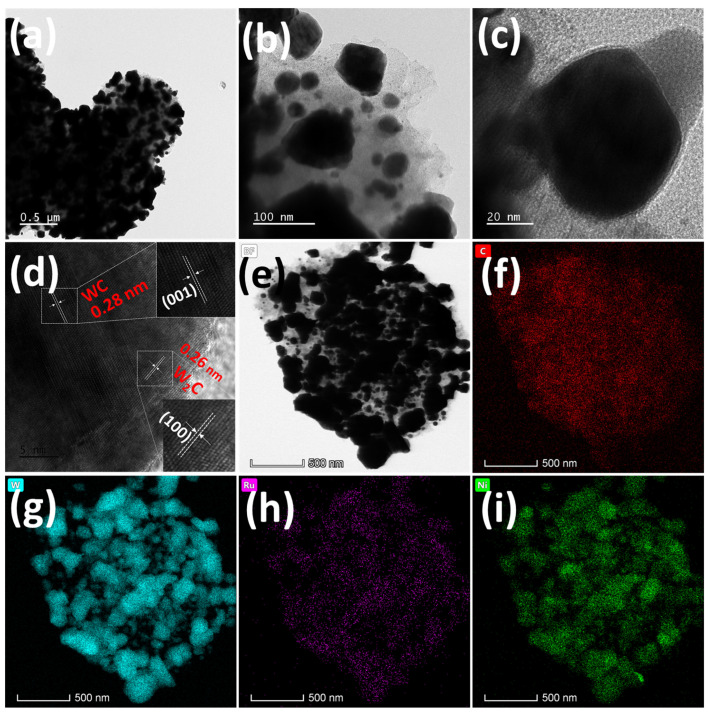
(**a**–**d**) HRTEM images and (**e**–**i**) the elemental mapping of WCx-RuNi.

**Figure 2:**
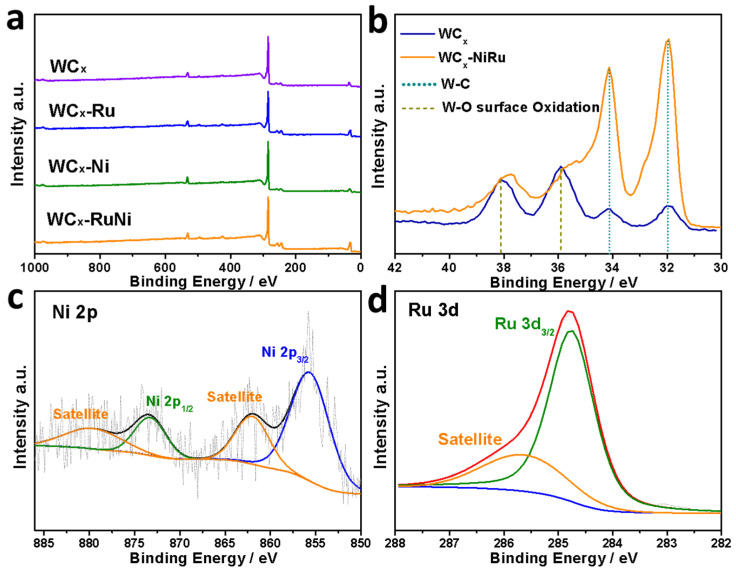
(**a**) XPS survey spectra, and high-resolution (**b**) W 4f, (**c**) Ni 2p, and (**d**) Ru 3d spectra of WCx-RuNi.

The HRTEM images (Figure 1a–d) reveal that the WCx-RuNi catalysts are composed of WCx nanocrystallites surrounded by carbon. The tungsten carbide nanoparticles as-synthesized exhibit a two-phase discontinuous microstructure consisting of WC and W_2_C, which display structural characteristics that are distinct from conventional tungsten carbide nanoparticles but resemble typical eutectoid structures. The interplanar distance of the WC lamellae is 0.28 nm, which is consistent with the (001) plane of WC [36]. Additionally, the neighboring W_2_C lamellae exhibit an interplanar distance of 2.60 nm, corresponding to the lattice spacing on the (100) plane of W_2_C [37]. Notably, lattice fringes attributed to RuNi alloy particles were not observed in the HRTEM images. This observation together with the XRD indicates that Ru and Ni are distributed as atomic states on the surface of WCx. Furthermore, the elemental mapping images (Figure 1e–i) demonstrate that carbon is uniformly distributed throughout the catalyst, while Ru, Ni, and W are similarly distributed, indicating uniform distribution of Ru and Ni atoms on the WCx support. The presence of Ni and Ru dual atoms anchored on the WCx surface contributes to their synergistic effect, ultimately enhancing the OER activity of the catalyst. X-ray photoelectron spectroscopy (XPS) was employed to investigate the surface composition, electronic structure, and surface oxidation states of the WCx-RuNi catalysts. The overall XPS spectrum in Figure 2a does not exhibit significant differences, and no distinct characteristic peaks of Ni and Ru are present due to their low contents. The W4f spectrum (Figure 2b) displays four primary characteristic peaks. The peaks at a binding energy of 31.90 and 34.11 eV are assigned to the W-C bond, while those at 35.88 and 38.11 eV are attributed to WOx in the surface passivating layer [38,39]. Compared to pure-phase WCx, the high-resolution W 4f spectra of WCx-RuNi exhibit surface oxygenation peaks at a reduced intensity. The high-resolution Ni 2p spectrum (Figure 2c) clearly shows two distinct characteristic peaks and satellite peaks at 855.78 and 873.44 eV, which correspond to Ni 2p_3/2_ and Ni 2p_1/2_, respectively [40,41,42]. In Figure 2d, the peak at 284.7 eV corresponds to Ru 3d_2/3_ [43,44,45]. These findings suggest that Ni and Ru are present in the WCx-RuNi catalysts at relatively low concentrations. This observation is in line with the results of HRTEM.nm

### 2.2. OER Electrocatalytic Performance

**Figure 3:**
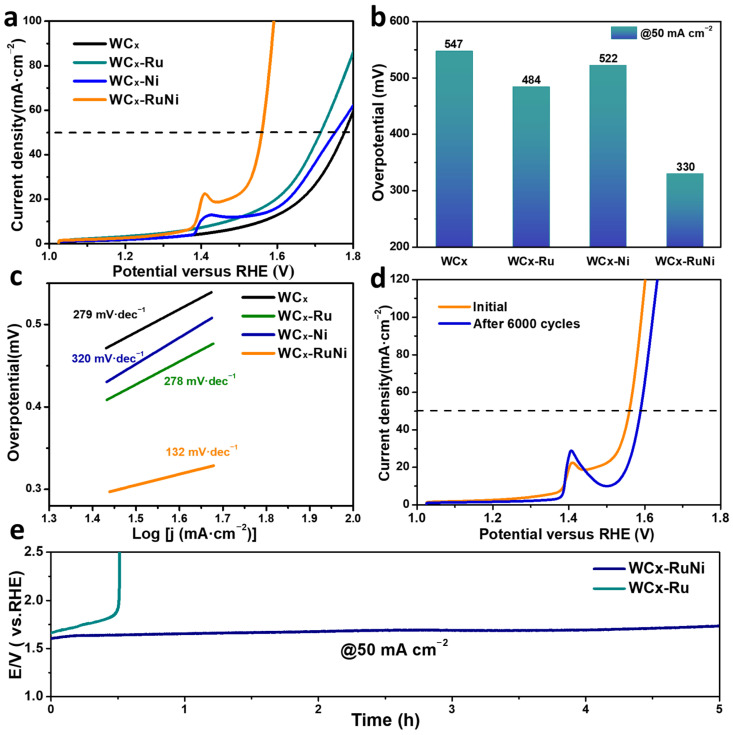
(**a**) LSV polarization curves of WCx, WCx-Ni, WCx-Ru, and WCx-NiRu; (**b**) overpotential columnar diagram with current density up to 50 mA/cm^2^; (**c**) Tafel slope of WCx, WCx-Ni, WCx-Ru and WCx-RuNi; (**d**) 6000 CV cycles; (**e**) chronopotentiometry of WCx-Ru and WCx-RuNi at a current density of 50 mA·cm^−2^.

We comparatively studied the electrocatalytic OER activities of WCx, WCx-Ni, WCx-Ru, and WCx-RuNi catalysts using linear sweep voltammetry (LSV). Figure 3a,b illustrates that WCx, WCx-Ni, and WCx-Ru require potentials of 547, 484, and 522 mV, respectively, to achieve a current density of 50 mA·cm^−2^. However, the WCx-RuNi samples exhibit the lowest potential of 330 mV, which is significantly lower than those of WCx-Fe, WCx-Ni, and bare WCx and is even better than that of commercial RuO_2_ (Figure S3, 343 mV). Such catalytic performance can match with or exceed many reported OER catalysts (Table S1). This enhanced OER activity can be attributed to the synergistic effect of Ru and Ni atoms uniformly distributed on the WCx surface under electrochemical conditions. We also prepared and tested WCx-RuFe, WCx-RuMn, and WCx-RuCo catalysts to test their electrocatalytic properties (Figure S4). At 50 mA·cm^−2^, the required potentials are 424, 502, and 428 mV, respectively. However, their activities are still lower than that of WCx-RuNi. Notably, the Tafel slope of WCx-RuNi (Figure 3c) is significantly lower (132 mV·dec^−1^) compared to WCx (279 mV·dec^−1^), WCx-Ni (320 mV·dec^−1^), and WCx-Ru (278 mV·dec^−1^). This result indicates the presence of faster reaction kinetics and electron transfer rates and is also mainly due to the synergistic effect of bimetallic atoms on WCx. As observed in the Nyquist plots (Figure S5), WCx-RuNi demonstrates a smaller semicircle diameter compared to WCx-Ru, indicating minimal charge-transfer resistance during the OER process. The durability of WCx-RuNi was evaluated by recording LSV curves after 6000 CV cycles. Interestingly, the potential only decreased by about 25 mV (Figure 3d). The durability of the catalysts was also verified via chronopotentiometry at a current density of 50 mA·cm^−2^ (Figure 3e), and WCx-RuNi showed high activity lasting 5 h, which was significantly superior to WCx-Ru. The results indicate the long-term stability of WCx-RuNi in the OER. Therefore, WCx loaded with binary metal single atoms has great potential for improving OER activity.

### 2.3. Catalytic Mechanism

**Figure 4:**
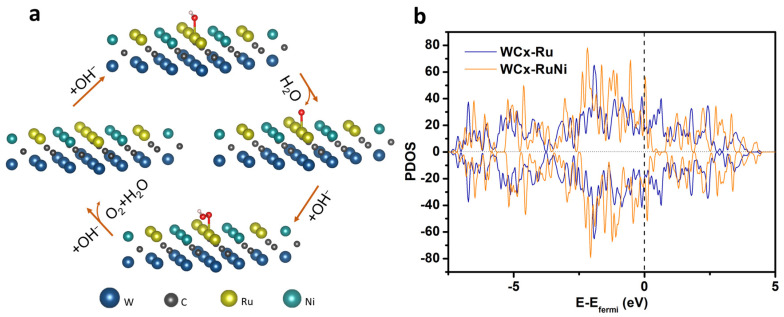
(**a**) The reaction paths on WCx-RuNi. (**b**) Total PDOS of WCx-RuNi and WCx-Ni.

A diagram of the OER process based on WCx-RuNi is shown in Figure 4a. To better understand the synergistic effect of Ni and Ru dual single atoms anchored on the WCx surface, we theoretically calculated the PDOS of WCx-RuNi and WCx-Ni (Figure 4b). Previous research has focused on single-atom-doped carbon, which shows limited catalytic activity and durability. However, well-crystallized WC nanocrystallites have emerged as promising catalysts for the OER. The introduction of Ni leads to an enhanced state density of WCx-RuNi near the Fermi level, which can enhance the adsorption of WCx-RuNi to oxygen-containing intermediates of related substances, and has stronger electron exchange. The center of the d band is closer to the Fermi level, meaning that d electrons are more likely to transmit to the valence band or from the conduction band to the d band. This result may indicate that there is a strongly coupled electron-lattice interaction that results in charge transfer between the d electron and the valence or conduction band. Moreover, WCx-RuNi exhibits better catalytic performance compared to WCx-Ni [46].

## 3. Conclusions

The NiRu dual-single atoms supported by WCx nanocrystals were prepared through a heat-treatment process, and exhibited excellent OER activity with an overpotential of 330 mV at 50 mA/cm^2^. This enhanced activity can be attributed to the synergistic effect of Ni and Ru on the WCx support. The addition of Ni to WCx-RuNi increases the state density near the Fermi level, and enhances the adsorption of oxygen-containing intermediates and promotes electron exchange. The higher proximity of the d band center to the Fermi level indicates a strong interaction between the d electrons and the valence or conduction band, facilitating charge transfer. This result highlights the crucial role of tungsten carbide as an ideal carrier material for high-performance OER catalysis, utilizing the metal–metal interaction with the WCx surface to stabilize metal atoms.

## Data Availability

Not applicable.

## Supplementary Materials

The following supporting information can be downloaded at: https://www.mdpi.com/article/10.3390/molecules28207040/s1, Figures S1–S3: Containing XRD, SEM and polarization curves. Figure S4: (a) LSV polarization curves of WCx-RuNi, WCx-RuFe, WCx-RuCo and WCx-RuMn, (b) Overpotential columnar diagram with current density up to 50 mA·cm^−2^. Figure S5: Nyquist plots of WCx-Ru and WCx-RuNi. Table S1: Comparison of the OER performance of reported catalysts.
