# Supplementary material for: WCx-Supported RuNi Single Atoms for Electrocatalytic Oxygen Evolution
Source: Molecules. 2023 Oct 12;28(20):7040. doi: 10.3390/molecules28207040 (PMC10609438; doi:10.3390/molecules28207040)
Supplement: Supplementary file 1 [file molecules-28-07040-s001.zip › molecules-2607414-supplementary.pdf]

WCx-Supported RuNi Single Atoms for Electrocatalytic Oxygen Evolution

Jirong Bai [1], Yaoyao Deng [1], Yuebin Lian [1], Quanfa Zhou [1,2] , Chunyong Zhang [2,*] and Yaqiong Su [3,*]

[1] Research Center of Secondary Resources and Environment, School of Chemical Engineering and Materials, Changzhou Institute of Technology, Changzhou 213022, China; baijr@czu.cn (J.B.); dengyy@czu.cn (Y.D.); lianyb@czu.cn (Y.L.); labzqf@czu.cn (Q.Z.)

[2] School of Chemistry and Environmental Engineering, Jiangsu University of Technology, Changzhou 213001, China

[3] School of Chemistry, Xi'an Key Laboratory of Sustainable Energy Materials Chemistry, State Key Laboratory of Electrical Insulation and Power Equipment, Xi'an Jiaotong University, Xi'an 710049, China

* Correspondence: zhangcy@jsut.edu.cn (C.Z.); yqsu1989@xjtu.edu.cn (Y.S.)

Experimental Section

Synthesis of WCx-RuNi, WCx-Ni, WCx-Ru, and WCx catalysts

First, a 0.1 M dopamine solution was prepared by dissolving 5 mmol of dopamine hydrochloride (DA) in a beaker containing 50 ml of DI water. The pH of the dopamine solution was adjusted to ~ 2 by adding about 2 ml of a 1 M HCl solution. Subsequently, 0.25 mmol RuCl3, 0.25 mmol MCl_x (M=Ni, Mn, Co, Fe), and 0.1 M Na2WO4·2H2O solution (50 ml) were added to the dopamine solution in succession under continuous stirring. The reaction mixture was stirred for another hour, and then the product was collected by centrifugation. The product was then washed with DI water and ethanol 3 times. Subsequently, the product was dried in an oven at 60 ℃ for 2 days to obtain the precursor, labeled as DA-WCx-RuM.

Then the precursor was carbonized in an argon furnace at 900 ℃ for 2 h, with a ramp of 3 ℃/min. After that, the black powder was naturally cooled to room temperature, and the sample was collected and donated as WCx-RuNi. For comparison, the same synthetic approach as that of WCx-RuNi was carried out to synthesize WCx, without the addition of metal chloride.

Electrochemical Measurements

Electrochemical testing for OER was implemented on a CHI760 electrochemical workstation (CH Instruments) with three-electrode set-up controlled in 1 M KOH. Graphite rod and Ag/AgCl were chosen as the counter electrode and the reference electrode, respectively. The working electrode was prepared as follows: 5 mg of the electrocatalyst was mixed with 485 μl of isopropyl alcohol and 15 μl of Nafion ionomers (5 wt %), followed by sonication for 30 min. A polished glassy carbon (GC) disk as the working electrode in diameter of 5 mm was drop-cast with 21 μl of the ink and dried at room temperature. Liner sweep voltammograms (LSV) polarization curves for OER with 95% *iR*-compensation were collected in 1 M KOH at a scan rate of 5 mV s^{-1}. Electrochemical impedance spectroscopy (EIS) was carried out with the frequency ranging from 10 kHz to 0.1 Hz. ADT tests were also conducted for OER by CV cycling for 6,000 cycles. All the potentials were calibrated to the RHE potential calculated from the equation *E*RHE = *E*Ag/AgCl + (0.197 + 0.0591pH) V. The durability of the catalysts was also verified via chronopotentiometry (CP) at a current density of 50 mA cm^{-2} for 18000 s.

Physical characterizations

Scanning electron microscope (SEM, Regulus 8100, 30 KV) and transmission electron microscope (TEM, FEI, TALOS F200X, 200 kV) were used to observe the morphology of the sample. The X-ray diffraction (XRD) measurement was carried out on a Bruker AXS D8 Advance with Cu K*α* radiation. The XPS measurements were performed using a Thermo ESCALAB250 equipment with Al Ka beam source.

Theoretical Calculation

In our work, first-principles calculations were performed with the projector augmented wave (PAW) method based on DFT. The exchange-functional was treated using the generalized gradient approximation (GGA) of the Perdew-Burke-Ernzerhof (PBE) functional. The WCx surface was obtained by randomly removing C atoms on the WCx crystal surface. In our calculation, the cut-off energy of the plane-wave basis was set at 450 eV for optimizing calculations of atoms and cell optimization. And the Brillouin zone integration is performed using 3×3×1 Monkhorst and Pack k-point sampling for surface structures. The self-consistent calculations apply a convergence energy threshold of 10^{-5} eV, and the maximum Hellmann Feynman force for each ionic optimization step is 0.05 eV $Å^{-1}$. Besides, in our modeling, the vacuum spacing in a direction perpendicular to the plane of the catalyst was at least 12 Å, the spin polarization was considered in all calculations.

Supplementary Figures and Tables

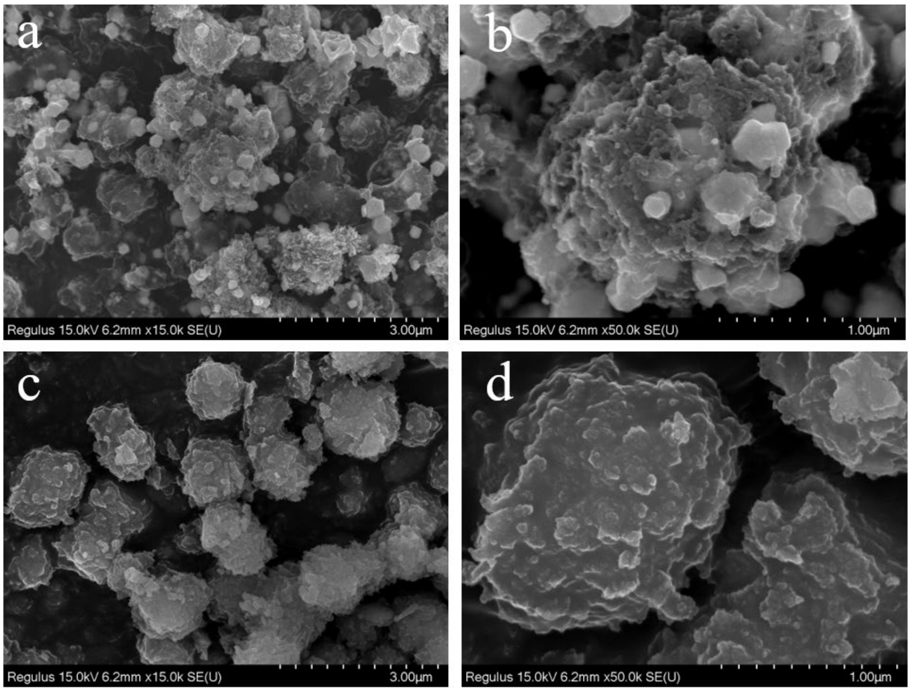

Figure S1 (a-b) SEM images of precursors, (c-d) SEM images of WC_x- RuNi

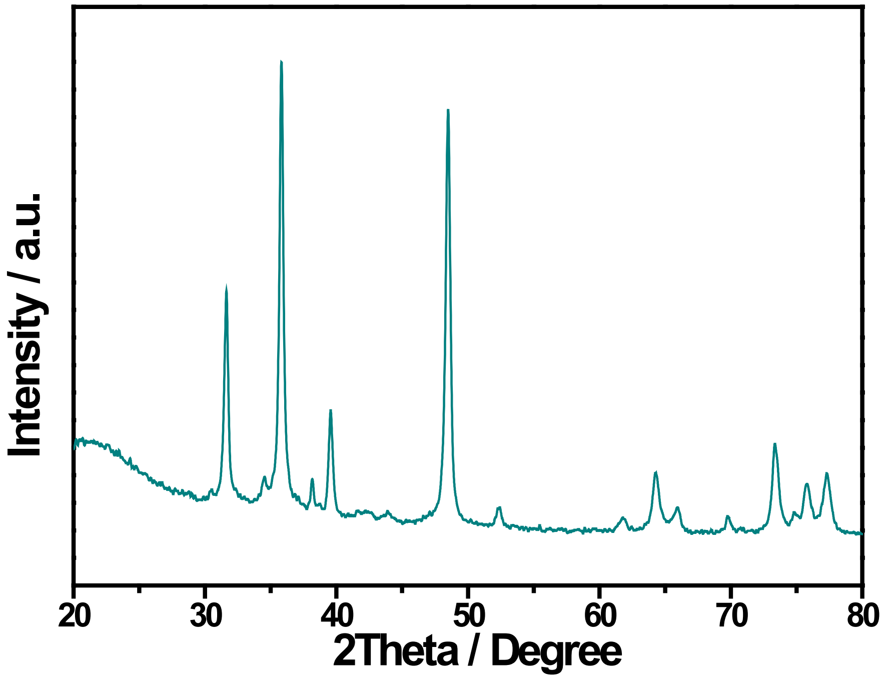

Figure S2 XRD patterns of WCx-RuNi catalysts

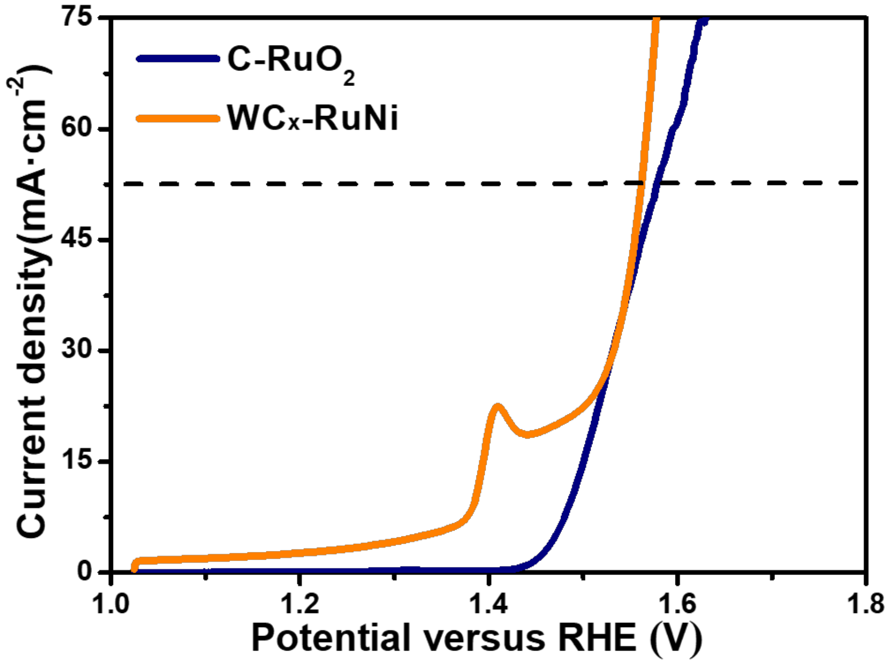

Figure S3 LSV polarization curves of C-RuO_2 and WCx-RuNi

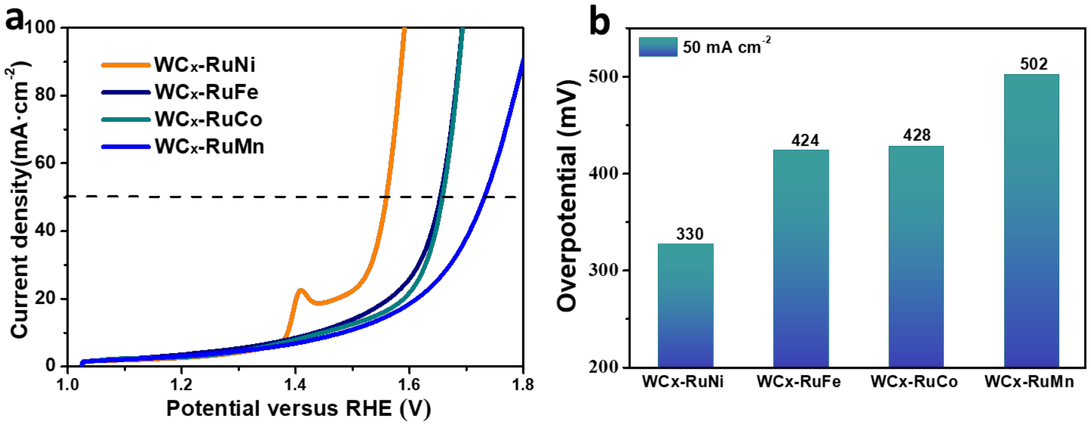

Figure S4 (a) LSV polarization curves of WC_x-RuNi, WC_x-RuFe, WC_x-RuCo and WC_x-RuMn, (b) Overpotential columnar diagram with current density up to 50 mA·cm^{-2}

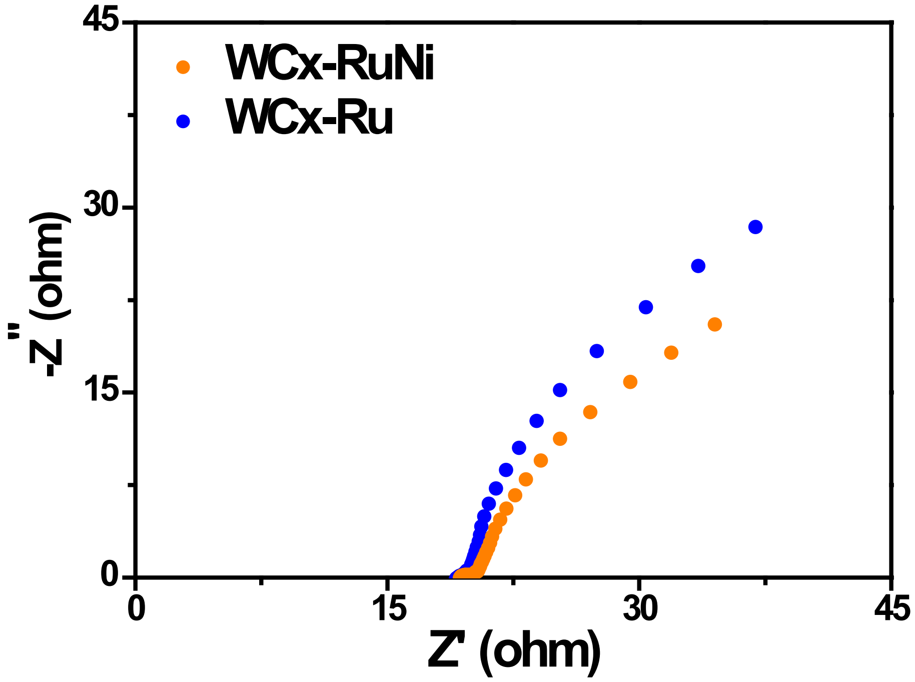

Figure S5 Nyquist plots of WCx-Ru and WCx-RuNi

Table S1 Comparison of the OER performance of reported catalysts

Catalysts	Eletrolyte	η@50 mA/cm^2	Reference
This work	1 M KOH	330	This work
RuCo/NC	1M KOH	300	Small, 2023, 19, e2207611
10%CeO_2–CoO	1M KOH	530	ACS Appl. Mater. Interfaces, 2021, 13, 46998-47009
NCO-2	1M KOH	340	Chem. Eng. J., 2022, 435, 134261
Co/CoO@COF	1M KOH	390	Nanoscale, 2021,13, 14854-14865
NiFe LDH	1.0 M KOH	360	App. Surf. Sci. 2023, 611, 155781
Cu_xS@NiMn LDH	1.0 M KOH	310	Int. J. Hydrogen Energ. 2023, 48, 1347-1359
